# Supplementary material for: Well-defined in-textile photolithography towards permeable textile electronics
Source: Nat Commun. 2024 Jan 30;15:887. doi: 10.1038/s41467-024-45287-y (PMC10828459; doi:10.1038/s41467-024-45287-y)
Supplement: Supplementary file 1 — Supplementary Information [file 41467_2024_45287_MOESM1_ESM.pdf]

Supplementary Information

Well-defined in-textile photolithography towards permeable textile electronics

Pengwei Wang[1,2], Xiaohao Ma[1,3], Zhiqiang Lin[1], Fan Chen[1], Zijian Chen[1], Hu Hong[1], Hailong Xu[1], Xinyi Zhang[3], Yuqing Shi[1,3], Qiyao Huang[1,4]*, Yuanjing Lin[3]*, Zijian Zheng[1,2,4,5]*

1. School of Fashion and Textiles, The Hong Kong Polytechnic University, Hong Kong SAR, China
2. Department of Applied Biology and Chemical Technology, The Hong Kong Polytechnic University, Hong Kong SAR, China
3. School of Microelectronics, Southern University of Science and Technology, Shenzhen 518055, China
4. Research Institute for Intelligent Wearable Systems, The Hong Kong Polytechnic University, Hong Kong SAR, China
5. Research Institute for Smart Energy, The Hong Kong Polytechnic University, Hong Kong SAR, China

*Corresponding email: qihuang@polyu.edu.hk, linyj2020@sustech.edu.cn, zijian.zheng@polyu.edu.hk

This PDF file includes:

Supplementary Figures 1 to 17

Supplementary Table 1

Supplementary References

Supplementary Figures

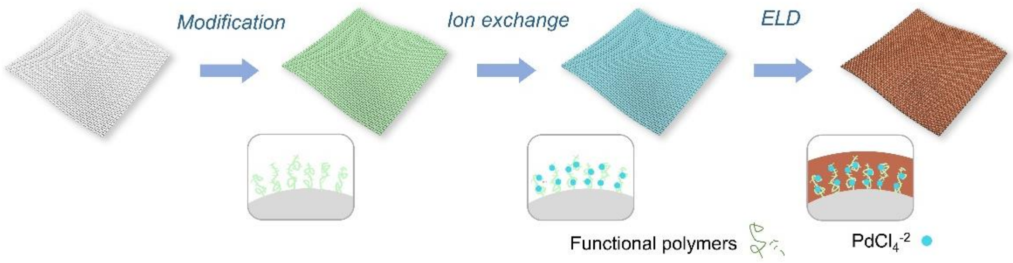

Supplementary Fig. 1. Schematic diagram of the PAMD process.

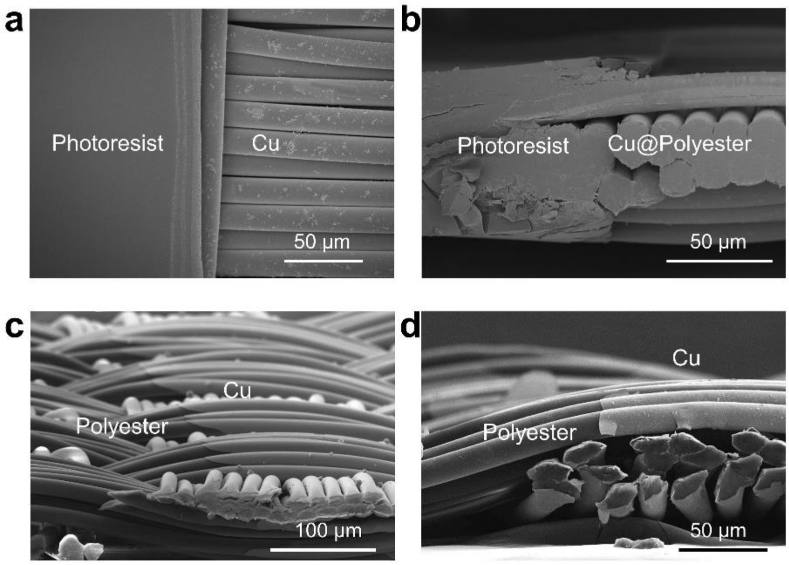

Supplementary Fig. 2. SEM images of photoresist-coated and Cu patterned polyester fabrics. **a-b** SEM image and cross-sectional SEM image of the photoresist-coated Cu-polyester fabric after the developing process. **c-d** Cross-sectional images showing the boundary of the Cu patterns in polyester fabrics after the etching process.

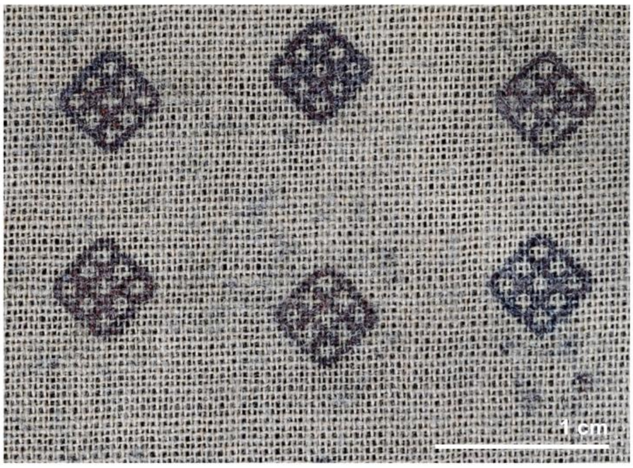

Supplementary Fig. 3. Digital image of the Cu electrode patterns in cotton fabric.

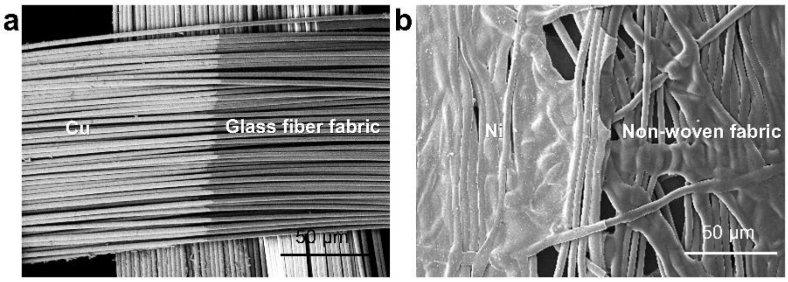

Supplementary Fig. 4. SEM images of conductive metal patterns in glass-fiber fabric and non-woven PP fabric. **a** SEM image of the Cu pattern on glass-fiber fabric. **b** SEM image of the Ni pattern on non-woven PP fabric.

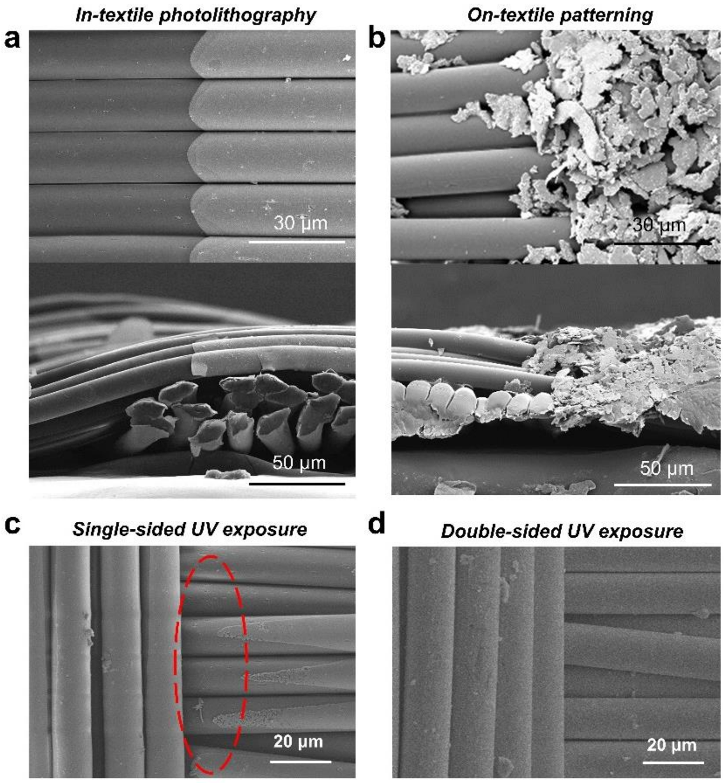

Supplementary Fig. 5. Cu pattern on *Polyester*$_{0.94}$ made by different patterning methods. **a** SEM image of the metal-textile boundary by in-textile photolithography. **b** SEM image of the metal-textile boundary by on-textile patterning method (screen printing). **c** Cross-sectional SEM image of the metal-textile boundary by single-sided UV exposure during the in-textile photolithography method. **d** Cross-sectional SEM image of the metal-textile boundary by double-sided UV exposure during the in-textile photolithography.

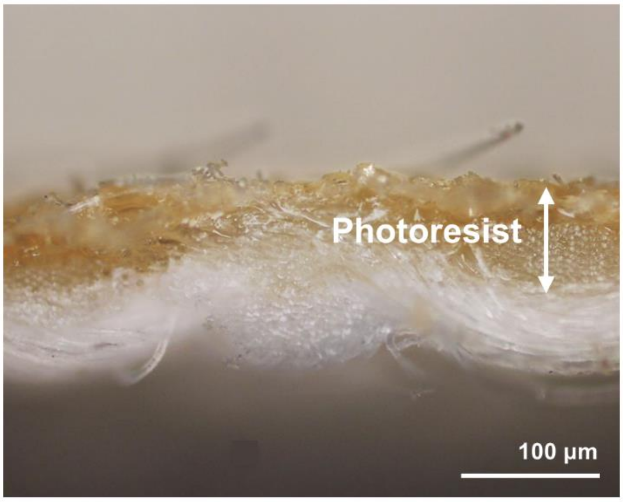

Supplementary Fig. 6. Cross-sectional optical image of the thicker Polyester textile fabricated via single-sided exposure. (textile with a thickness of 170 μm and photoresist with a thickness of 80 μm)

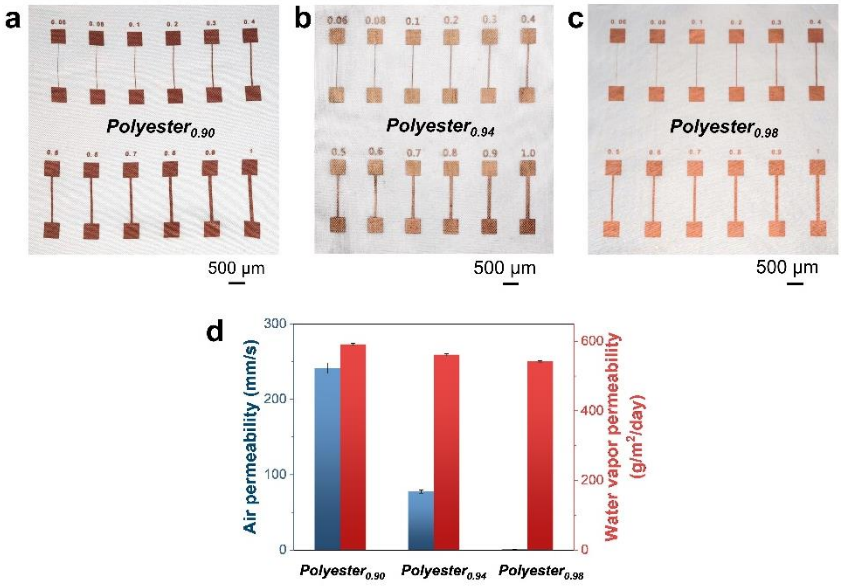

Supplementary Fig. 7. Cu patterns with different widths in polyester fabrics with different yarn coverages and their permeability performance. **a-c** Digital images showing the Cu patterns with different linewidths pattered in Polyester$_{0.90}$ fabric, Polyester$_{0.94}$ fabric, and Polyester$_{0.98}$ fabric. (0.90, 0.94, and 0.98 are the cover factors of the polyester fabrics). **d** Air permeability and water vapor permeability of the polyester fabrics with different cover factors. Error bars represent the s.d. of the mean from three fabrics coated with Cu.

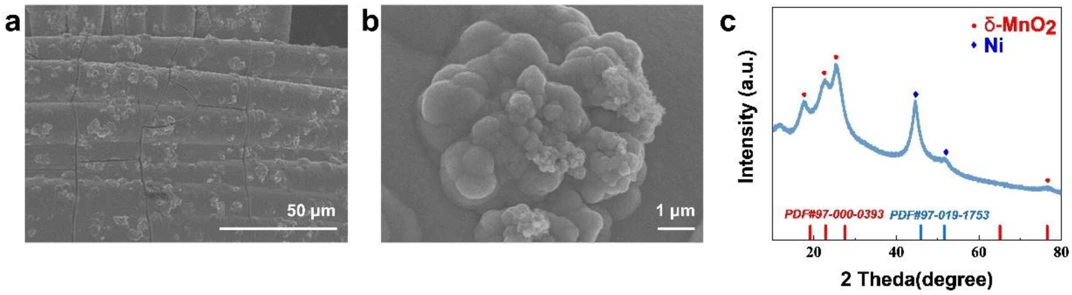

Supplementary Fig. 8. Characterization of MnO_2 deposited on Ni interdigital electrodes. **a-b** SEM image of deposited MnO_2. **c** X-ray diffraction (XRD) characterization of MnO_2@Ni.

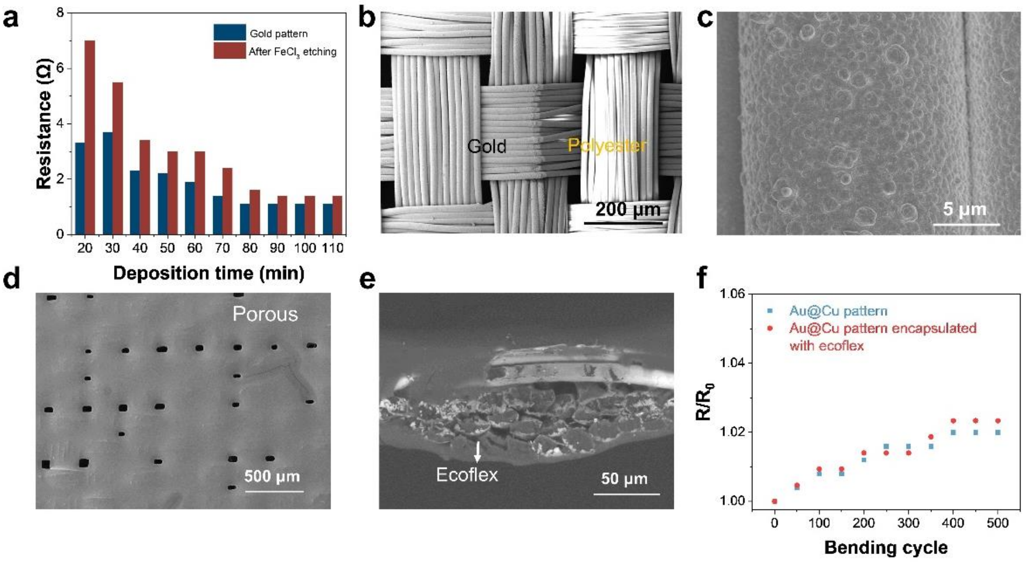

Supplementary Fig. 9. Characterization of the Au@Cu circuit in polyester fabric. **a** Resistance of the samples with different Au deposition times and $FeCl_3$ etching times. **b** SEM image of the Au@Cu-textile boundary. **c** Micro-structure of the Au coating. **d** SEM image of the Ecoflex encapsulation coating on interconnect regions. **e** Cross-sectional SEM image of the Ecoflex encapsulation coating on interconnect regions. **f** Resistance change of the Au@Cu pattern and Ecoflex-coated Au@Cu pattern.

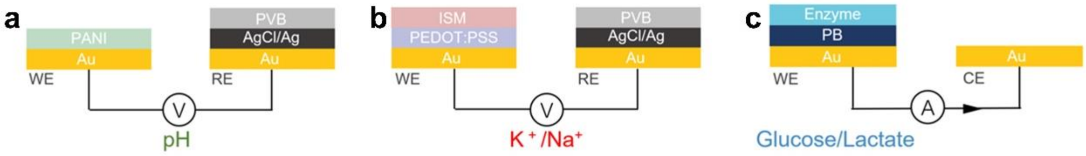

Supplementary Fig. 10. Structure of the electrodes of three types of sweat sensors. **a** pH sensor. **b** K^+/Na^+ sensor. **c** Glucose/Lactate sensor.

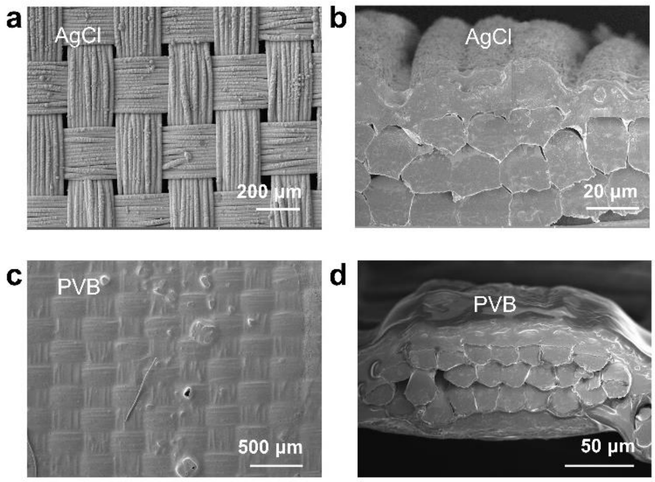

Supplementary Fig. 11. Characterization of Ag/AgCl/PVB reference electrode. **a** SEM image of the metal-textile boundary by in-textile photolithography. **b** SEM image of the metal-textile boundary by screen printing. **c** Cross-sectional SEM image of the metal-textile boundary by in-textile photolithography. **d** Cross-sectional SEM image of the metal-textile boundary by screen printing.

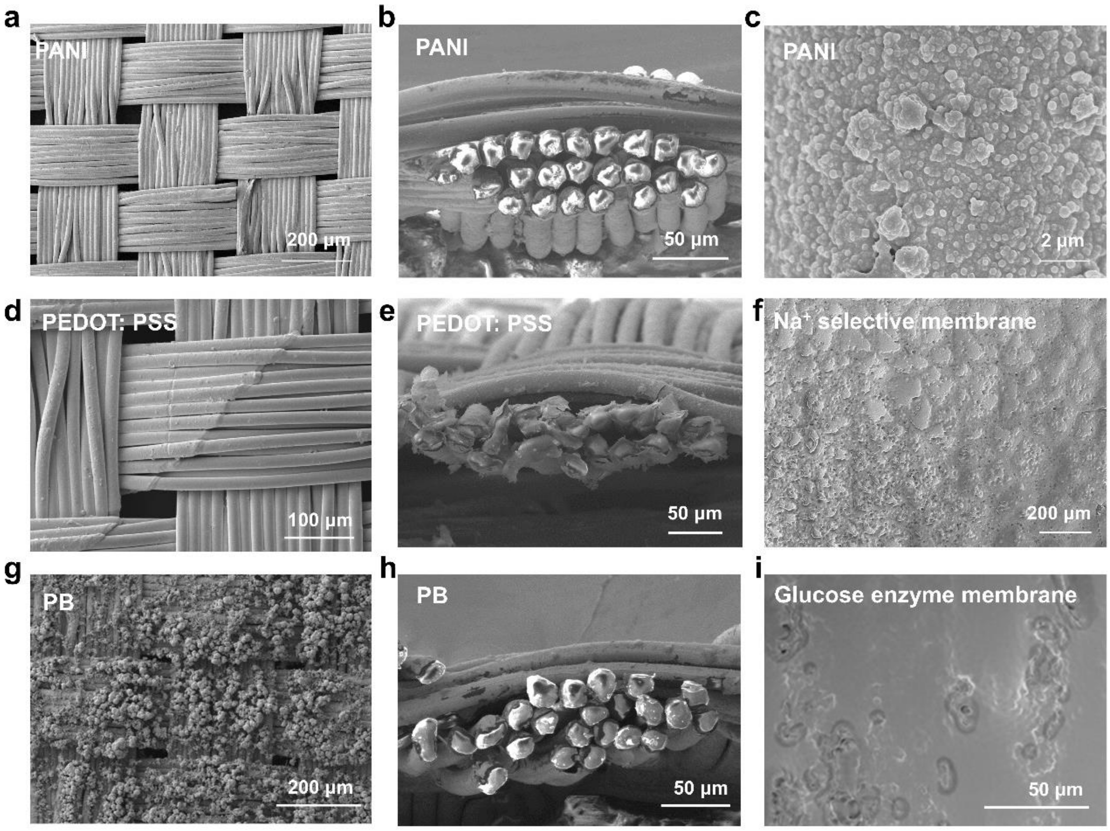

Supplementary Fig. 12. SEM images of sweat sensors. **a** PANI coating for pH sensor. **b** Cross-sectional image of the PANI. **C** Micro-structure of the PANI coating. **d** PEDOT: PSS coating for Na^+/K^+ sensors. **e** Cross-sectional image of the PEDOT: PSS. **f** Ion selective membrane of the Na^+ sensors. **g** PB coating for Glucose/lactate sensors. **h** Cross-sectional image of the PB. **i** Enzyme membrane of the glucose sensors.

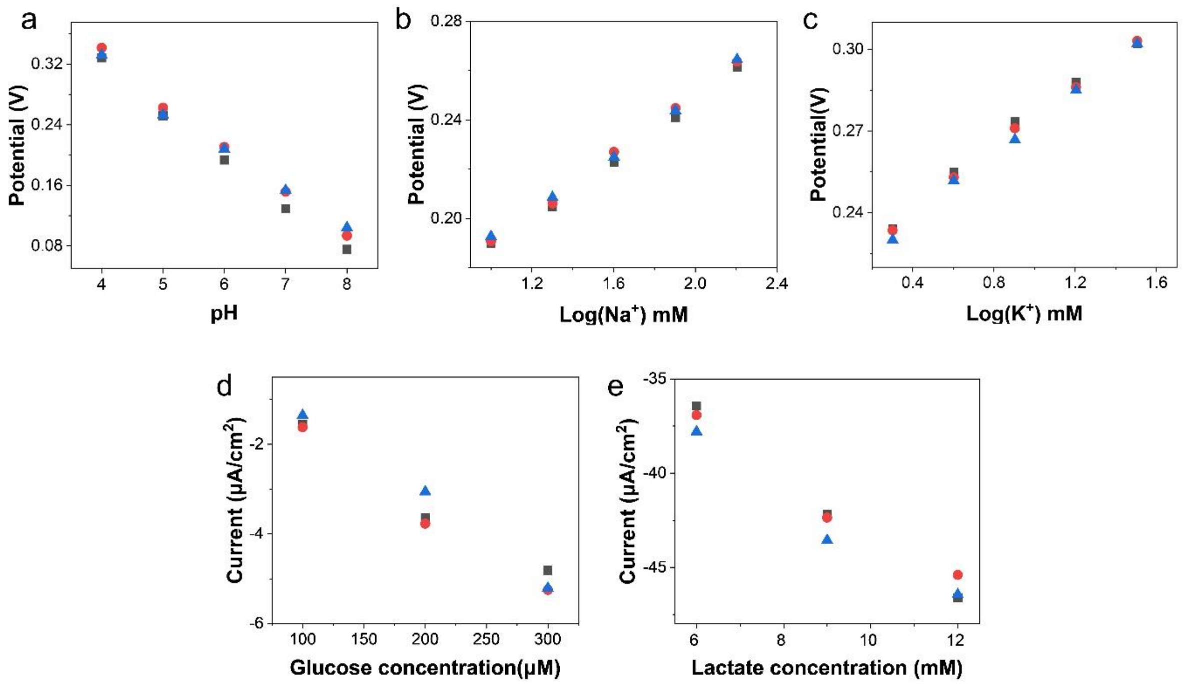

Supplementary Fig. 13. Reproducibility of the textile-based sensors. **a** pH sensors. **b** Na^+ sensors. **c** K^+ sensors. **d** Glucose sensors. **e** Lactate sensors.

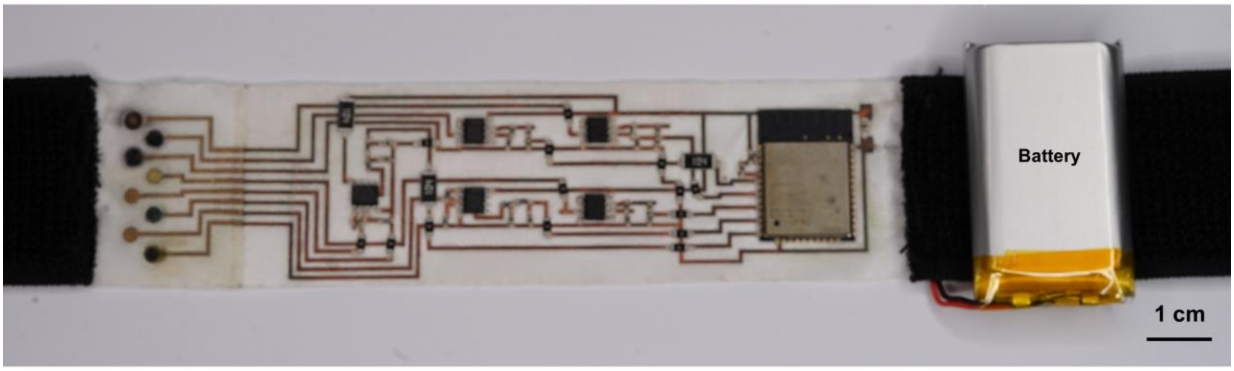

Supplementary Fig. 14. Digital image of the in-textile headband with a commercial Li-ion battery.

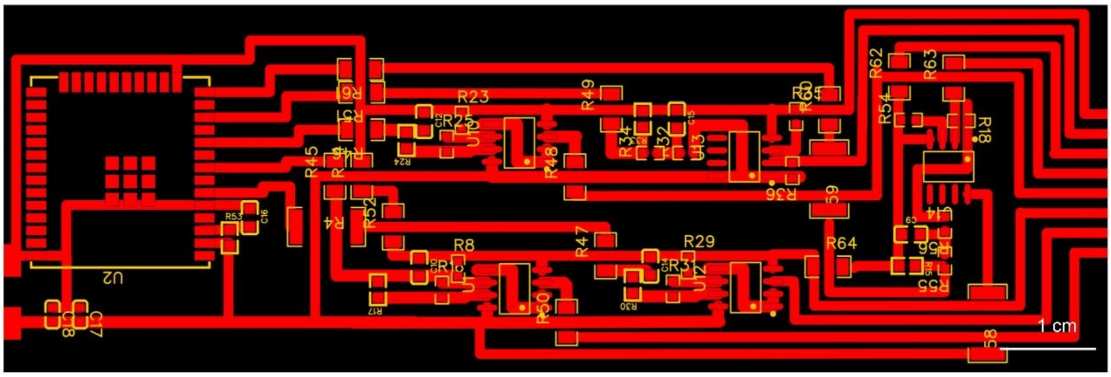

Supplementary Fig. 15. One-layer circuit design based on *Polyester*$_{0.94}$ fabric (3.5 cm*10 cm).

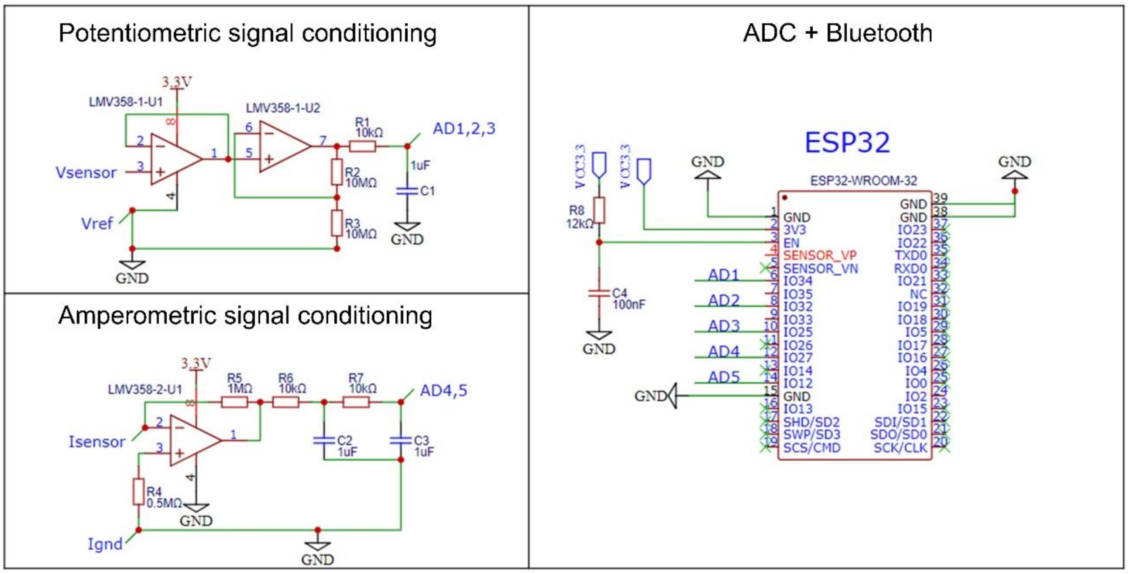

Supplementary Fig. 16. Schematic diagram of signal-conditioning (potentiometric and amperometric) and wireless circuit. (Bluetooth, ADC part).

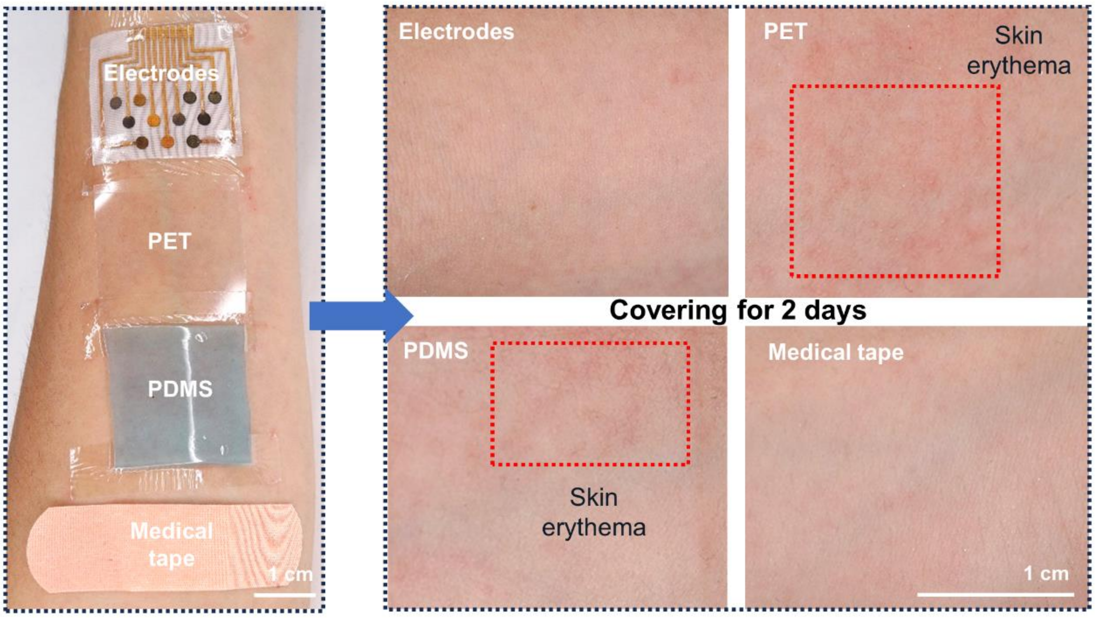

Supplementary Fig. 17. Biocompatibility of the sensor electrodes showing the skin irritation results of the forearm of a volunteer which was covered with the sensor electrodes, polyester (PET) film, PDMS film, and commercial medical tape.

Supplementary Tables

Supplementary Table 1. Comparison of the patterning resolution and electrical conductivity of conductive patterns developed by different patterning technologies reported in the literature.

Materials	Patterning techniques	Resolution	Resistance/conductivity	Ref.
CNT/PU	Screen printing	1.5 cm	0.2 kΩ/sq	1
CNT@Silk	Stitching	-	310 S/cm	2
Ag/AgCl/TPU	Screen printing	1 mm	5,000 S/cm	3
Ag flake	Screen printing	500 µm	1,333 S/cm	4
Plated Cu	Electroless plating	2.5 mm	0.5 Ω/sq	5
Ag flake/TPU	Screen printing	1 mm	4.31×10^4 S/cm	6
Liquid metal	Embroidery	635 µm	4.2 Ω/m	7
AgNW/TPU	Laser scribing	135 µm	5,030 S/cm	8
Cu	In-textile photolithography	100 µm	1.7 Ω/cm	This work

CNT: carbon nanotube; PU: polyurethane; AgNW: silver nanowire; TPU: thermoplastic polyurethane.

Supplementary References

1. Langley DP*, et al.* Metallic nanowire networks: effects of thermal annealing on electrical resistance. *Nanoscale* **6**, 13535-13543 (2014).
2. Yin Z*, et al.* Splash-resistant and light-weight silk-sheathed wires for textile electronics. *Nano Lett* **18**, 7085-7091 (2018).
3. Yokus MA, Foote R, Jur JS. Printed stretchable interconnects for smart garments: design, fabrication, and characterization. *IEEE Sens J* **16**, 7967-7976 (2016).
4. Jin H, Matsuhisa N, Lee S, Abbas M, Yokota T, Someya T. Enhancing the performance of stretchable conductors for E-textiles by controlled ink permeation. *Adv Mater* **29**, 1605848 (2017).
5. Wills KA*, et al.* Additive process for patterned metallized conductive tracks on cotton with applications in smart textiles. *J Text Inst* **109**, 268-277 (2018).
6. Suikkola J*, et al.* Screen-printing fabrication and characterization of stretchable electronics. *Sci Rep* **6**, 25784 (2016).
7. Lin RZ*, et al.* Digitally-embroidered liquid metal electronic textiles for wearable wireless systems. *Nat Commun* **13**, 2190 (2022).
8. Yao SS, Yang J, Poblete FR, Hu XG, Zhu Y. Multifunctional electronic textiles using silver nanowire composites. *ACS Appl Mater Inter* **11**, 31028-31037 (2019).
