# Supplementary material for: Well-defined in-textile photolithography towards permeable textile electronics
Source: Nat Commun. 2024 Jan 30;15:887. doi: 10.1038/s41467-024-45287-y (PMC10828459; doi:10.1038/s41467-024-45287-y)
Supplement: Supplementary file 3 — Description of additional supplementary files [file 41467_2024_45287_MOESM3_ESM.pdf]

DESCRIPTION OF ADDITIONAL SUPPLEMENTARY FILES DOCUMENT

Supplementary Movie 1. A wearable temperature monitoring patch with an in-situ alarming function.

Supplementary Movie 2. On-body real-time perspiration analysis on a subject's forehead
